# Delayed anemia assessment in patients treated with oral artemisinin derivatives for uncomplicated malaria: a pooled analysis of clinical trials data from Mali

**DOI:** 10.1186/1475-2875-13-358

**Published:** 2014-09-12

**Authors:** Issaka Sagara, Renaud Piarroux, Abdoulaye Djimde, Roch Giorgi, Kassoum Kayentao, Ogobara K Doumbo, Jean Gaudart

**Affiliations:** Malaria Research and Training Center, Department of Epidemiology of Parasitic Diseases, Faculty of Medicine and Odonto-Stomatogy, University of Sciences, Techniques and Technologies of Bamako, BP 1805, Point G Bamako, Mali; Aix-Marseille University, UMR912 SESSTIM (INSERM, IRD, AMU), 13005 Marseille, France; Aix-Marseille University, UMR MD3, 13005 Marseille, France

**Keywords:** Uncomplicated, *Plasmodium falciparum*, Malaria, Delayed anaemia, Artemisinin, Clinical trials

## Abstract

**Background:**

In sub-Saharan Africa, artemisinin-based combination therapy (ACT) and injectable artesunate are the first-line treatments for uncomplicated and severe *Plasmodium falciparum* malaria, respectively. However, recent studies suggest that delayed anaemia is associated with these treatments in non-immune travellers. This paper aimed to assess the risk factors associated with delayed anaemia after falciparum malaria treatment with artemisinin-containing drugs in malaria-endemic populations.

**Methods:**

Pooled, individual malaria patient data were extracted from 13 clinical trials performed from 2002 to 2011 in various settings of Mali. Treatment regimens were artemether-lumefantrine, artesunate plus amodiaquine, artesunate plus sulphadoxine-pyrimethamine, artesunate plus sulphamethoxypyrazine-pyrimethamine, artesunate plus mefloquine, artesunate-pyronaridine, artesunate monotherapy, chloroquine, sulphadoxine-pyrimethamine, amodiaquine and sulphadoxine-pyrimethamine plus amodiaquine. Univariate and multivariate analyses were performed using the generalized linear and latent mixed model procedures to assess risk factors associated with haemoglobin concentration evolution and anaemia during the treatment follow-up.

**Results:**

A total of 5,990 participants were recruited and followed from day 0 to day 28. The participants’ median age was five years, ranging from three months to 70 years. There was a decrease in haemoglobin level on day 7 in all treatment arms, but the magnitude varied across treatments. There was a significant risk of haemoglobin level decrease on day 7 in the artemisinin-based therapy compared to the non-artemisinin treatments. The risk of haemoglobin concentration drop was associated with age group < five years old (0.61 g/dL 95% CI (0.71 to 0.51), p < 0.001), baseline high parasite density (0.43 g/dL 95% CI (0.51 to 0.35), p < 0.001) and treatment failure (0.40 g/dL 95% CI (0.59 to 0.20), p = 0.018), while high haemoglobin level at baseline was a protective factor (0.53 to 0.59) p < 0.001). No association was found between artemisinin-based therapy and severe delayed anaemia.

**Conclusions:**

Oral artemisinin derivative treatments for uncomplicated *P. falciparum* malaria are associated with a transient and clinically moderate haemoglobin decrease by day 7 but not associated with a delayed severe anaemia.

**Electronic supplementary material:**

The online version of this article (doi:10.1186/1475-2875-13-358) contains supplementary material, which is available to authorized users.

## Background

According to the World Health Organization (WHO) 2013 report, 207 million cases of malaria were estimated to have occurred in 2012 with 627,000 deaths [1]. Early diagnosis and treatment of malaria reduce disease progression, prevent deaths and contribute to reduce malaria transmission [2]. To counter the threat of resistance of *Plasmodium falciparum* to monotherapy, and to improve treatment outcome, WHO recommends the use of artemisinin-based combination therapy (ACT) for the treatment of uncomplicated *P. falciparum* malaria [3], and the injectable artesunate as the first-line treatment of severe malaria [4]. However, recent data, mainly from case reports raised concerns about possible delayed anaemia associated with artemisinin derivative treatments [5–9]. WHO recognized that post-treatment haemolytic anaemia was not specific to a particular injectable artesunate formulation and that anaemia was also described following the use of intramuscular artemether and oral artemether-lumefantrine [10, 11]. Therefore, WHO called for the systematic monitoring of haemoglobin after malaria treatment initiation up to one month.

There is limited large-scale data in the literature from malaria-endemic areas assessing haematological parameters, particularly anaemia among patients treated with the artemisinin-containing malaria treatments. Recently a publication from a large-scale, pooled, multi-country study data from malaria-endemic areas assessed haematologic parameter changes in patients treated with ACT (artesunate-amodiaquine, artesunate plus sulphadoxine-pyrimethamine, dihydro-artemisinin-piperaquine and artemether-lumefantrine) and other therapy (artesunate, amodiaquine and sulphadoxine-pyrimethamine plus amodiaquine) [12]. That study found more anaemia adverse events in the artemisinin-based therapy compared to the non-artemisinin therapy at the 28^th^ day of follow-up, but did not provide the specific day of the occurrence of anaemia during the follow-up period. As precise knowledge of the specific occurrence day of haemoglobin change or anaemia occurrence is of clinical relevance, this paper aimed to perform a pooled analysis of data from 13 clinical trials conducted in Mali. The goal of this pooled data analysis was to describe the dynamics of haemoglobin levels, delayed anaemia and the associated risk or protective factors during each follow-up day, up to 28 days.

## Methods

### Data source

A pooled, malaria-treatment, clinical trial analysis was performed, using individual data extracted from clinical trial databases of 13 trials conducted by researchers from Malaria Research and Training Centre (MRTC), Bamako, a research institution within the University of Sciences, Techniques and Technologies of Bamako (USTTB), Mali. The different studies were performed from 2002 to 2011 in various malaria-transmission settings of Mali [13–23] (Ouologuem D *et al.* Host immunological factors involved in clearance of drug resistant falciparum malaria, unpublished observation; Maiga H *et al.* Dhfr-dhps quadruple mutant predicts sulfadoxine-pyrimethamine resistance in Mali, an emerging sulfadoxine-pyrimethamine resistance setting, unpublished observation).

### Treatment regimens

The different malaria treatment regimens were: (i) ACT: artemether-lumefantrine ((AL) Coartem® from Novartis); artesunate plus amodiaquine ((AS-AQ), loose or fixed-dose combination, Arsucam/Coarsucam®, both from Sanofi-Aventis); artesunate plus sulphadoxine-pyrimethamine ((AS-SP), loose combination; AS, Arsumax® from Sanofi-Aventis and SP, Fansidar® from Roche); artesunate plus sulphamethoxypyrazine-pyrimethamine ((AS-SMP), loose combination, Coarinate® from Dafra Pharma); artesunate plus mefloquine ((AS-MEF), loose combination, Artequin® from Mepha Pharma); and, artesunate-pyronaridine ((AS-PYR), fixed-dose combination, Pyramax® from Shin Poong); (ii) artesunate oral monotherapy ((AS), Arsumax® or Asunate Denk® respectively from Sanofi-Aventis and Denk Pharma); (iii) non ACT: chloroquine (CQ); amodiaquine (AQ); sulphadoxine-pyrimethamine (SP) and sulphadoxine-pyrimethamine plus amodiaquine ((SP-AQ), loose combination).

Treatment courses durations were three days for all except for: a) SP given as a single dose; and, b) AS given up to five days in one study [21] and seven days in another study [13]. The treatment doses were given by the study investigators and details are provided in each study reference [13–23] (Ouologuem D *et al.* Host immunological factors involved in clearance of drug resistant falciparum malaria, unpublished observation; Maiga H *et al.*: Dhfr-dhps quadruple mutant predicts sulfadoxine-pyrimethamine resistance in Mali, an emerging sulfadoxine-pyrimethamine resistance setting, unpublished observation) and also in a Additional file 1.

### Outcome definitions

Data were systematically analysed according to three outcomes: dynamic of haemoglobin level, haemoglobin drop and anaemia occurrence.

Haemoglobin levels were analysed as a continuous variable (haemoglobin changes outcome) and as categorical variables (haemoglobin drop and anaemia occurrence). Drop of haemoglobin value was defined from day 0 to the day of the different follow-up visit (day 7, 14, 28) with the following thresholds: ≥1 g/dL or ≥2 g/dL. Anaemia was defined as a haemoglobin value <10 g/dL and severe anaemia as haemoglobin value <8 g/dL as described elsewhere [12]. Delayed anemia was defined as an anaemia (severe or not) observed any time of follow-up from day 7 to day 28 [5].

For these outcomes, multivariate analyses assessed the different associated risk factors during each visit after treatment (day 7, 14 or 28) in order to determine any significant changes during the follow-up. These analyses included all participants for whom haemoglobin data were available on day 0 and at least one follow-up day (day 7 or 14 or 28). Specifically for the delayed severe anaemia occurrence, associated risk factors were assessed with different time points: (i) from day 7 to day 28; (ii) from day 14 to day 28; and, (iii) on day 28. These analyses used a subset of participants for which the haemoglobin data were available for all follow-up visits (day 0, 7, 14, and 28). Different late or delayed severe anaemia periods were assessed: (i) an absence of severe anaemia on day 0 followed by its occurrence during any subsequent days for follow-up (7 or 14 or 28); (ii) an absence of severe anaemia on day 0 and day 7 followed by its occurrence during any subsequent days for follow-up (i.e., day 14 or 28); and, (iii) an absence of severe anaemia on day 0 and day 7 and day 14 followed by its occurrence on day 28.

### Statistical analysis

Descriptive statistics provided the total number of participants, means, standard deviations, medians, minimums, and maximums for quantitative variables and proportions for qualitative variables.

Univariate analyses assessed the haemoglobin changes between day 0 and the day of post-treatment evaluation (day 7, 14 or 28) using paired Student test in each treatment arm.

For both continuous variable of haemoglobin changes and categorized variable of haemoglobin drop or anaemia outcomes, multivariate analyses were performed using generalized linear and latent mixed model (GLLAMM), with study site as a latent factor and as random effect. Models were systematically adjusted for covariates such as age, parasite density at baseline (log transformed), malaria treatment, and treatment failure during the follow-up. For the haemoglobin changes outcome, the gaussian family (with the identity canonical link) was used to estimate the coefficients of covariate effects. For the categorized haemoglobin drop and anaemia outcomes, the binomial family (the logit canonical link) was used to estimate odds ratios associated with covariates.

The confidence intervals (CIs) were estimated at 95% and p value <0.05 was considered significant. Data were analysed with GLLAMM procedure using Stata Software version 12.1 (Stata Corp).

### Ethical issues

Each study had a prior approval from the institutional ethical committee at the Faculty of Medicine, Pharmacy and Odonto-Stomatogy (FMPOS)/USTTB, Bamako, Mali. Written consent was obtained in each study from each participant or parent/legal guardian.

## Results

### Study information and baseline characteristics

**Figure 1:**
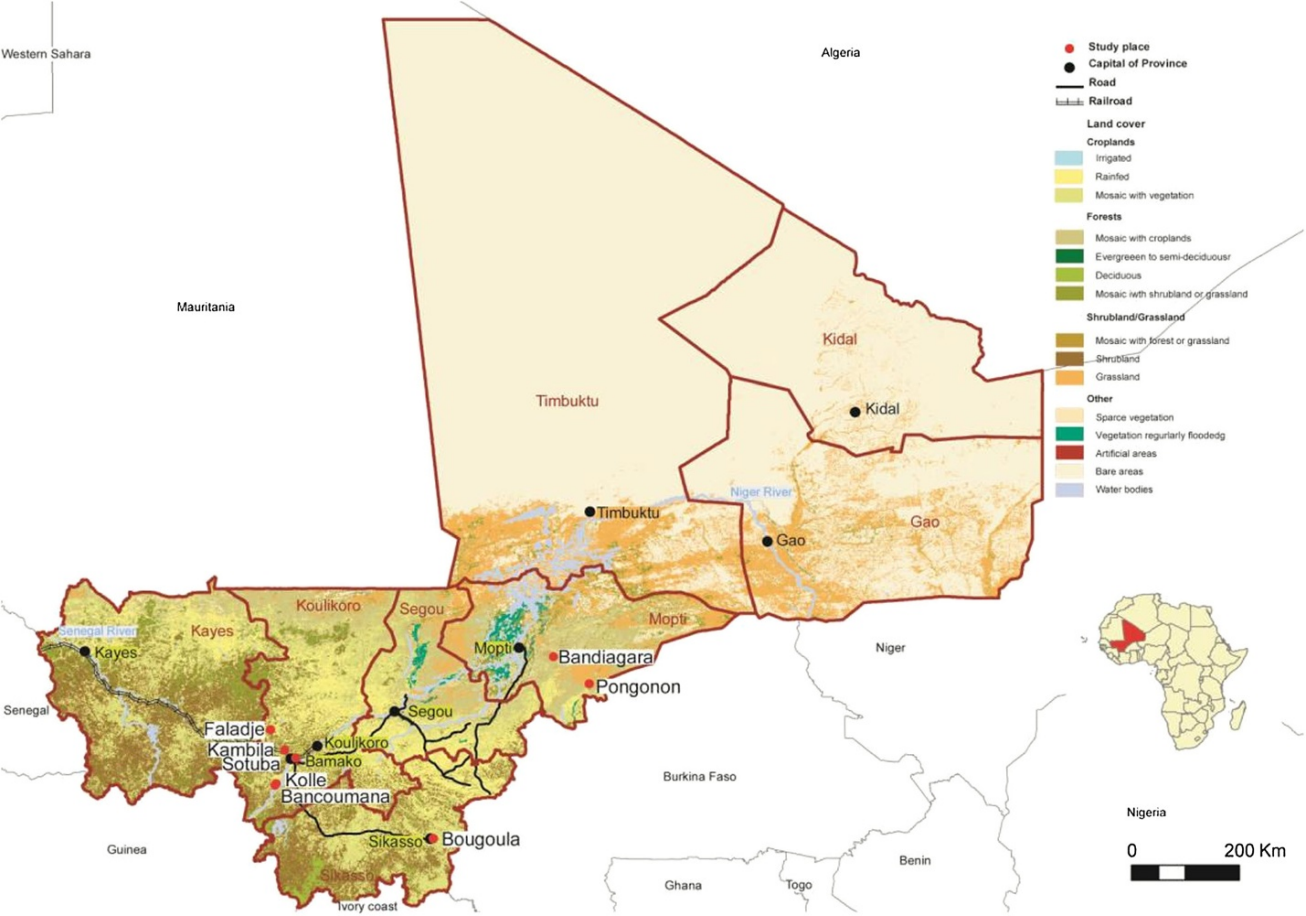
**Study sites.** Study sites are indicated by red dots.

This pooled data of 13 trials in eight Malian sites (Figure 1) enrolled 5,990 participants with various malaria treatments (Table 1), including ACT (AL, AS-AQ, AS-SP, AS-SMP, AS-MEF, AS-PYR; oral AS monotherapy; and, non ACT (CQ, AQ , SP and SP-AQ).

**Table 1:** **Number of patients per study and treatment arm**

Study* (year)	Treatment											Total
	AL	AS-AQ	AS-PY	AS-SP	AS-SMP	AS-MF	AS	SPAQ	SP	AQ	CQ	
[13] (2010–11)							100					100
UO (2009)	337											337
[14] (2007–08)	44		86									130
[15] (2007)	66		132									198
[16] (2006)	68	135										203
[17] (2005–07)	257	255		259								771
[18] (2005)		133		134				133				400
[19] (2004–05)	235					235						470
[20] (2003–04)	303				303							606
[21] (2002–04)		252		249			251					752
UO (2002–04)											948	948
UO (2002–03)									619			619
[22] (2002–03)									121	115	220	456
Total	1,310	775	218	642	303	235	351	133	740	115	1,168	5,990

*The studies are cited in descending order from conduct year ranging from 2011 to 2002. Numbers in the first column represent publication references as cited in the text. UO, unpublished observations.

Table 2 shows that the median age for the pooled data was five years, ranging from three months to 70 years. The baseline (day 0) proportion of anaemia was 36% and the baseline parasite density geometric mean was 17,789 parasites/μl.

**Table 2:** **Baseline characteristics and scheduled hemoglobin evaluation visit per study and site**

Study*	Site	N	Haemogobin evaluation days	Age median [min, max]	*P. f.*	Anaemia prev.
[13] (2010–11)	Bougoula- Hameau	100	0, 3, 14, 28	6 [1,11]	28,027	48%
UO (2009)	Kolle	77	0, 14, 28	6 [1,18]	44,492	55%
	Faladje	88		6 [1,14]	47,942	26%
	Bandiagara	100		7 [1, 61]	32,703	28%
	Pongonon	72		5 [1, 34]	18,841	43%
[14] (2007–08)	Bougoula- Hameau	130	0, 3, 7, 28	6 [2,10]	32,430	31%
[15] (2007)	Bougoula- Hameau	198	0, 3, 7, 28	11 [6, 52]	22,115	11%
[16] (2006)	Bancoumana	203	0, 7, 14, 28	5 [0.9, 24]	22,183	50%
[17] (2005–07)	Bougoula- Hameau	771	0, 7, 14, 28	4 [0.5, 69]	20,463	50%
[18] (2005)	Faladje	400	0, 14, 28	36 [6, 60]	23,705	48%
[19] (2004–05)	Kambila	470	0, 14, 28	6 [1,70]	18,258	13%
[20] (2003–04)	Sotuba	606	0, 28	11 [0.6, 63]	13,592	14%
[21] (2002–04)	Bougoula- Hameau	752	0, 3, 7, 14, 28	3 [0.6, 56]	15463	44%
UO (2002–04)	Kolle	655	0, 7, 14	7 [0.5, 64]	12200	25%
	Bandiagara	293		6 [0.7, 30]	12686	29%
UO (2002–03)	Bougoula- Hameau	619	0, 14, 28	3 [0.5, 65]	14897	42%
[22] (2002–03)	Kolle	456	0, 7, 14, 28	3 [0.3, 6]	19778	51%
Total		5,990		5 [0.3, 70]	17,789	36%

Note: *P. f., Plasmodium falciparum* geometric mean*;* Anaemia prev. (dichotomous variable), anaemia prevalence. Anaemia was defined as haemoglobin value <10.0 g/dL; Numbers in first column represent publication references as cited in the text.

*The studies are citied in descending order from conduct year ranging from 2011 to 2002. The number given is their publication reference as applicable.

UO, unpublished observations.

### Assessment of haemoglobin level per visit day

On day 7: overall, there was an average haemoglobin decrease of 0.4 g/dL compared to day 0. Univariate analyses showed a significant decrease (p < 0.001) in all treatments except for AQ (Table 3). The frequency of haemoglobin drop to ≥1 g/dL and to ≥2 g/dL compared to day 0 was 32.2% (858/2,668) and 11.4% (303/2,668), respectively.

**Table 3:** **Haemoglobin changes from baseline to day of follow-up after treatment per treatment arm**

	Day 0-day 7				Day 0-day 14				Day 0-day 28			
	N	Mean 0	Mean 7	Mean diff,	N	Mean 0,	Mean 14,	Mean diff,	N	Mean 0,	Mean 28,	Mean diff,
		[SD]	[SD]	(P-value)		[SD]	[SD]	P-value		[SD]	[SD]	(P-value)
AL	193	10.6 [1.7]	10.2 [1.7]	−0.4 **(<0.001)**	861	10.5 [1.9]	10.7 [1.6]	+0.2 **(0.015)**	890	10.7 [1.8]	11.3 [1.6]	+0.6 **(<0.001)**
AS-AQ	403	10.1 [1.8]	9.7 [1.7]	−0.4 **(<0.001)**	729	10.0 [1.7]	10.2 [1.5]	+0.2 **(<0.001)**	667	10.0 [1.8]	10.7 [1.7]	+0.7 **(<0.001)**
AS-PYR	218	11.4 [1.5]	11.0 [1.5]	−0.4 **(0.001)**	--	--	--	--	213	11.4 [1.5]	11.9 [1.3]	+0.4 **(<0.001)**
AS-SP	272	10.2 [1.9]	9.7 [1.7]	−0.5 **(<0.001)**	630	10.0 [1.8]	10.1 [1.6]	+0.1 **(0.023)**	610	10.0 [1.8]	10.7 [1.5]	+0.7 **(<0.001)**
AS-SMP	--	--	--	--	--	--	--	--	105	11.8 [1.8]	12.0 [1.5]	+0.2 (0.096)
AS-MEF	--	--	--	--	228	11.4 [1.5]	11.2 [1.3]	−0.2 **0.032**	213	11.5 [1.5]	11.9 [1.1]	+0.3 **(0.0003)**
AS	348	10.2 [1.7]	9.5 [1.5]	−0.7 **(<0.001)**	348	10.2 [1.7]	10.3 [1.4]	+0.1 (0.198)	297	10.2 [1.8]	11.0 [1.5]	+0.8 **(<0.001)**
SP-AQ	--	--	--	--	133	10.0 [1.6]	10.4 [1.5]	+0.4 **(0.001)**	131	10.0 [1.6]	11.1 [1.5]	+1.1 **(<0.001)**
SP	114	10.0 [1.8]	9.4 [1.5]	−0.6 **(<0.001)**	679	10.2 [1.8]	10.3 [1.7]	+0.1 (0.102)	706	10.3 [1.8]	10.9 [1.7]	+0.6 **(<0.001)**
AQ	104	9.9 [1.5]	9.7 [1.5]	−0.2 (0.3)	62	9.7 [1.6]	9.9 [1.3]	+0.2 (0.251)	97	9.9 [1.5]	10.4 [1.6]	+0.5 **(0.002)**
CQ	1016	10.9 [1.9]	10.6 [1.7]	−0.3 **(<0.001)**	978	10.9 [1.9]	11.0 [1.7]	+0.1 (0.065)	162	9.86 [1.63]	10.01 [1.54]	+0.2 (0.261)

Note: Mean 0, Mean 7, Mean 14, Mean 28 are haemoglobin mean on days 0, 7, 14, and 28, respectively; SD, standard deviation of the mean;

Mean diff, haemoglobin mean on the day of the follow-up minus haemoglobin mean on day 0.

Multivariate analyses showed a higher risk for haemoglobin decrease in the ACT (AS or the combined AS-AQ/AS-SP/AS-PYR and AL) compared to the non ACT (CQ/AQ/SP). The risk of haemoglobin decrease was significant for the AS, mean decrease of 0.69 g/dL 95% CI (0.95-0.44), p < 0.001; and the AS-containing combinations: AS-AQ/AS-SP/AS-PYR, 0.34 g/dL, 95% CI (0.57-0.11), p = 0.003 (Table 4). A similar trend was observed for the haemoglobin drop to ≥1 g/dL for AS and the AS-containing combinations: AS-AQ/AS-SP/AS-PYR, but was only significant for the AS arm (OR = 1.69, 95%CI (1.31-2.18); p < 0.001) (Table 5). There was no risk associated with a haemoglobin drop ≥2 g/dL on day 7 with artemisinin-derivative treatments (Table 6). There was even a protective effect against haemoglobin drop ≥2 g/dL with AL (OR = 0.51, 95% CI (0.28-0.90); p = 0.02) and the AS-containing combinations: AS-AQ/AS-SP/AS-PYR (OR = 0.70, 95% CI (0.52-0.94); p = 0.02) compared to non-artemisinin treatments (Table 6).

**Table 4:** **Multivariate analysis assessing haemoglobin changes per treatment arm during different post-treatment follow-up visits**

	Day 7 (n=2,657)		Day 14 (n=4,638)		Day 28 (n=4,082)	
Covariates**	β* (95% CI)	*p*	β* (95% CI)	*p*	β* (95% CI)	*p*
AL	−0.21 (−0.48-0.05)	*0.11*	−0.06 (−0.17- 0.06)	*0.34*	−0.05 (−0.18- 0.08)	*0.47*
AS+partners	−0.34 (−0.57- -0.11)	***0.003***	−0.12 (−0.22- -0.02)	***0.02***	−0.03 (−0.14- 0.08)	*0.62*
AS	−0.69 (−0.95- -0.44)	***<0.001***	−0.03 (−0.18-0.11)	*0.68*	0.15 (−0.02-0.32)	*0.08*
<5 years old	−0.61 (−0.71- -0.51)	***<0.001***	−0.62 (−0.71- -0.54)	***<0.001***	−0.51 (−0.60- -0.42)	***<0.001***
Haemo day 0	0.56 (0.53-0.59)	***<0.001***	0.48 (0.46-0.50)	***<0.001***	0.45 (0.43-0.47)	***<0.001***
*P. f*. day 0	−0.43 (−0.51- -0.35)	***<0.001***	−0.33 (−0.40- -0.026)	***<0.001***	−0.04 (−0.12-0.04)	*0.29*
Treat fail	−0.40 (−0.59- -0.20)	***0.018***	−0.25 (−0.39- -0.11)	***<0.001***	−0.4 (−0.51- -0.29)	***<0.001***

Note: *β is the regression coefficient estimate obtained from the generalized linear latent and mixed models using site as random effect;

**Covariates references were: CQ, AQ, SP for day 7 and CQ, AQ, SP and SP-AQ for day 14 and day 28 for treatment, the age ≥5 years old for the age group, the 28-day cured parasite outcome after treatment for the treatment failure (Treat fail) outcome, the baseline parasite density was log transformed and treated as continuous variable; AS + partners were: AS-AQ, AS-SP, AS-PYR for day 7, AS-AQ, AS-SP, AS-MEF for day 14 and AS-AQ, AS-SP, AS-PYR, AS-SMP and AS-MEF for day 28.

**Table 5:** **Multivariate analysis estimating risk of haemoglobin drop from baseline to ≥1 g/dL during different post-treatment follow-up visits**

	Day 7 (2,657)		Day 14 (n=4,638)		Day 28 (n=4,082)	
	OR* (95% CI)	*p*	OR* (95% CI)	*p*	OR* (95% CI)	*p*
Covariates**						
AL	0.82 (0.58-1.17)	*0.27*	1.03 (0.82-1.30)	*0.81*	1.10 (0.82-1.48)	*0.52*
AS+partners	1.15 (0.95-1.39)	*0.16*	0.93 (0.75-1.14)	*0.47*	1.0 (0.77-1.29)	*0.99*
AS	1.69 (1.31-2.18)	***<0.001***	0.97 (0.71-1.32)	*0.84*	1.01 (0.68-1.50)	*0.95*
<5 years old	0.90 (0.76-1.06)	*0.21*	0.79 (0.68-0.92)	***0.003***	0.72 (0.60-0.88)	***0.001***
*P. f*. day 0	1.56 (1.33-1.83	***<0.001***	1.60 (1.38-1.87)	***<0.001***	1.02 (0.85-1.23)	*0.81*
Treat fail	1.77 (1.24-2.55)	***0.002***	1.49 (1.14-1.94)	***0.003***	1.67 (1.32-2.11)	***<0.001***

Note: *OR is the odds ratio obtained from generalized linear and latent mixed model with binomial family using site as random effect;

**Covariates references were: CQ, AQ, SP for day 7 and CQ, AQ, SP and SP-AQ for day 14 and day 28 for treatment, the age ≥5 years old for the age group, the 28-day cured parasite outcome after treatment for the treatment failure (Treat fail) outcome, the baseline parasite density was log transformed and treated as continuous variable; AS + partners were: AS-AQ, AS-SP, AS-PYR for day 7, AS-AQ, AS-SP, AS-MEF for day 14 and AS-AQ, AS-SP, AS-PYR, AS-SMP and AS-MEF for day 28.

**Table 6:** **Multivariate analysis estimating risk of haemoglobin drop from baseline to ≥2 g/dL during different post-treatment follow-up visits**

	Day 7 (2,657)		Day 14 (n=4,638)		Day 28 (n=4,082)	
	OR* (95% CI)	*p*	OR*(95% CI)	*p*	OR* (95% CI)	*p*
Covariates**						
AL	0.51 (0.28-0.90)	***0.02***	1.33 (0.95-1.87)	*0.10*	0.86 (0.54-1.39)	*0.55*
AS+partners	0.70 (0.52-0.94)	***0.02***	0.95 (0.69-1.32)	*0.78*	0.96 (0.63-1.44)	*0.83*
AS	1.36 (0.97-1.92)	*0.08*	0.99 (0.61-1.59)	*0.96*	1.07 (0.58-1.96)	*0.83*
<5 years old	0.87 (0.68-1.11)	*0.25*	0.82 (0.65-1.04)	*0.10*	0.82 (0.60-1.12)	*0.21*
*P.f.* day 0	1.80 (1.41-2.30	***<0.001***	1.68 (1.33-2.12)	***<0.001***	1.30 (0.96-.75)	*0.09*
Treat fail	1.43 (0.88-2.32)	*0.15*	1.63 (1.13-2.38)	***0.01***	2.03 (1.44-2.87)	***<0.001***

Note: *OR is the odds ratio obtained from generalized linear and latent mixed model with binomial family using site as random effect;

**Covariates references were: CQ, AQ, SP for day 7 and CQ, AQ, SP and SP-AQ for day 14 and day 28 for treatment, the age ≥ 5 years old for the age group, the 28-day cured parasite outcome after treatment for the treatment failure (Treat fail) outcome, the baseline parasite density was log transformed and treated as continuous variable; AS + partners were: AS-AQ, AS-SP, AS-PYR for day 7, AS-AQ, AS-SP, AS-MEF for day 14 and AS-AQ, AS-SP, AS-PYR, AS-SMP and AS-MEF for day 28.

A significant risk of haemoglobin decrease was associated with other covariates (Table 4) such as the age group < five years (p < 0.001), a high parasite density at baseline (p < 0.001), a treatment failure (p = 0.018) and a lower baseline haemoglobin level ( p < 0.001). There was also a significant risk of haemoglobin drop ≥1 g/dL and ≥2 g/dL for patients showing a high parasite density at baseline with OR of 1.56 (95% CI (1.33-1.83); p < 0.001) and 1.80 ((1.41-2.30); p < 0.001), respectively (Tables 5 and 6).

On day 14: overall, there was an average haemoglobin increase of 0.13 g/dL compared to day 0. Univariate analyses in Table 3 showed a relatively small increase of haemoglobin level on day 14 compared to day 0 in all treatment arms except for AS-MEF. The frequency of haemoglobin drop ≥1 g/dL and ≥2 g/dL compared to day 0 was 21.6% (1,004/4,648) and 8% (371/4,648), respectively.

The multivariate analysis showed no risk of haemoglobin decrease or haemoglobin drop ≥1 g/dL, or haemoglobin drop ≥2 g/dL associated with any of the malaria treatment arms on day 14 (Tables 4, 5 and 6). However, there was a significant risk of haemoglobin decrease or drop to ≥1 g/dL or to ≥2 g/dL with a high parasite density at baseline and the treatment failure (Tables 4, 5 and 6).

On day 28: overall, there was an average haemoglobin increase of 0.63 g/dL compared to day 0. Univariate analyses in Table 3 indicate that the increases were significant in all treatment arms except for CQ (p = 0.261) and AS-SMP (p = 0.096). The frequency of haemoglobin drop ≥1 g/dL and ≥2 g/dL compared to day 0 was 13.9% (569/4,091) and 4.9% (200/4,091), respectively.

Multivariate analyses showed no risk of haemoglobin decrease or haemoglobin drop ≥1 g/dL or haemoglobin drop ≥2 g/dL associated with any malaria treatment arms on day 28 (Tables 4, 5 and 6). However, there was a significant risk of haemoglobin decrease or drop ≥1 or ≥2 g/dL with treatment failure (Tables 4, 5 and 6), with the higher risk association for haemoglobin drop ≥2 g/dL (OR: 2.03, 95% CI (1.44-2.87), p < 0.001) (Table 6).

### Assessment of anaemia and severe anaemia

Anaemia (haemoglobin <10 g/dL) frequency on day 0, 7, 14, and 28 was 35.5% (2,115/5,957), 45.7% (1,221/2,672), 35.3% (1,640/4,652), and 23.9% (979/4,099), respectively.

The severe anaemia (haemoglobin <8 g/dL) frequency on day 0, 7, 14, and 28 was, respectively, 9.0% (536/5,957), 10.1% (269/2,672), 6.2% (286/4,652), and 4.0% (165/4,099).

**Figure 2:**
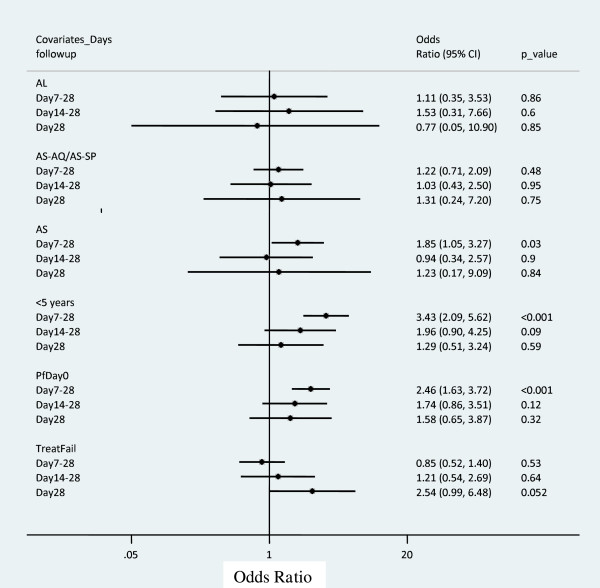
**Multivariate analysis estimating late severe anaemia occurrence risk among patients who participated to all haemoglobin evaluation from day 0, 7, 14, and 28.** n = 1,053, 956 and 941, respectively, for patients who participated to severe anaemia evaluation from day 7 to day 28, day 14 to day 28, and day 28. AL, artemether-lumefantrine; AS-AQ/AS-SP, artesunate plus amodiaquine/artesunate plus sulphadoxine-pyrimethamine; AS, artesunate; <5 years, <5 years old; PfDay0, Log *Plasmodium falciparum* density on day 0; TreatFail, Treatment failure outcome. Odds ratio were obtained from generalized linear and latent mixed model with binomial family using site as random effect; Covariates references were: CQ, AQ and SP for treatment, the age ≥5 years old for the age group, the 28-day cured parasite outcome after treatment for the treatment failure outcome, the baseline parasite density was log transformed and treated as continuous variable.

Among a total of 1,053 eligible patients (with no severe anaemia on day 0 and haemoglobin data available on days 0, 7, 14, and 28), 135 patients (12.8%) experienced severe anaemia from day 7 to day 28. Multivariate analysis showed a risk of severe anaemia associated with the AS arm from day 7 to day 28 (OR = 1.85 (1.05-3.27); p = 0.03) compared to the non-artemisinin-contained treatments (CQ alone/AQ alone/SP alone/SP-AQ) (Figure 2). But, no risk was present with the other artemisinin-derivative treatments (AL, AS-AQ/AS-SP).

Multivariate analyses showed no risk of severe anaemia associated with artemisinin-derivative treatment arms (AS, AL, AS-AQ/AS-SP) compared to the non-artemisinin-contained treatments (CQ alone/AQ alone/SP alone/SP-AQ) from day 7 to day 28 (Figure 2).

Among a total of 956 eligible patients (with no severe anaemia before day 14 and haemoglobin data available on days 0, 7, 14, and 28), 38 patients (4%) experienced severe anaemia from day 14 to day 28. Multivariate analyses (Figure 2) showed no risk of severe anaemia associated with artemisinin-derivative treatment arms (AS, AL, AS-AQ/AS-SP) compared to the control non-artemisinin-contained treatments (CQ alone/AQ alone/SP alone/SP-AQ) from day 14 to day 28.

Among a total of 941 patients (with no severe anaemia before day 28 and haemoglobin data available on days 0, 7, 14, and 28), 23 patients (2.4%) experienced severe anemia on day 28. Multivariate analyses (Figure 2) showed no risk of severe anaemia associated with the artemisinin-derivative treatment arms (AS, AL, AS-AQ/AS-SP) compared to the control non-artemisinin-contained treatments (CQ alone/AQ alone/SP alone/SP-AQ) on day 28.

The risk of blood transfusion was assessed: a total of five cases of blood transfusion were documented, all in children ≤ three years old. Transfusion was done based both on low haemoglobin and clinical anaemia symptoms as judged by the physician. There were three in AS-AQ arm (one on day 2, one on day 3 and one on day 31), one in AS arm (on day 7) and one in SP arm (unknown day). Data of these patients were excluded from this analysis once they received the blood transfusion. The transfusion information was not available for one study (Maiga H *et al.* Dhfr-dhps quadruple mutant predicts sulfadoxine-pyrimethamine resistance in Mali, an emerging sulfadoxine-pyrimethamine resistance setting, unpublished observation) conducted in 2002–2004, which enrolled 948 patients in CQ arm.

## Discussion

The study found no evidence of delayed anaemia risk associated with oral artemisinin derivatives. However, a moderate haemoglobin decrease or drop on day 7 was found. There were also risk factors associated with anaemia or haemoglobin decrease, such as treatment failure or high parasite density.

There are limited large-scale data addressing specifically haemoglobin changes or anaemia occurrence after oral artemisinin-derivative treatments. Recently, WHO recognized post-treatment haemolytic anaemia with artemisinin derivatives [10, 11] and, therefore, called for the monitoring of haemoglobin as these data came from report cases from non-endemic malaria populations. This present study pooled individual data from 13 distinct studies involving 5,990 participants, of all age groups, from Mali, West Africa. The use of various artemisinin-containing treatments, including ACT and non-ACT offered the opportunity for comparison of haemoglobin changes or drop between different malaria treatments.

The baseline anaemia frequency was 36% and varied across the sites. It was lower than from a study [12], which reported an average frequency of 60% with pooled multi-country data, including part of data from Mali on artemisinin derivatives, which are also included in this study.

This study documented a transient and clinically moderate but significant decrease of haemoglobin on day 7 after treatment initiation in almost all malaria treatments. This finding was consistent with previous studies [24, 25]. Mechanisms of anaemia or haemoglobin decrease following malaria episode are multiple, and are not fully understood. Various causes include haemolysis from malaria itself or from parasitized and non-parasitized red cells by the immune system or even from auto haemolysis mechanism [26, 27]. Day 7 analysis, after adjusting for covariates (multivariate analysis) indicated that some ACT (AS or the combined AS-AQ/AS-SP/AS-PYR) compared to the non-ACT (CQ/AQ/SP) and covariates (age group < five years, high parasite density at baseline and treatment failure) were associated with higher haemoglobin decrease. Another study reported that younger age, high parasite density at baseline, and treatment failure were associated with the risk of post-treatment anaemia [12].

On day 14 and 28 there was significant increase in haemoglobin in most of treatment arms with maximum increase on day 28. The multivariate analyses indicated that ACT was not more associated with the risk of haemoglobin drop compared to non-ACT, while other covariates (such as treatment failure) were associated with the risk of haemoglobin drop. As reported in this study, haemoglobin increase or anaemia recovery, which is common by day 28, was also documented in several studies [12, 24, 25].

The frequency of severe anaemia, which was about 9% on day 0 and 10% on day 7, decreased gradually to 4% by day 28. Multivariate analyses indicated that there was a risk of severe anaemia associated with the AS arm from day 7 to day 28, compared to the non-ACT (CQ/AQ/SP/SP + AQ). But this was not evident from day 14 to 28 or on day 28. These observations could be explained by the relatively important decrease of haemoglobin shown in this study on day 7 particularly with AS arm (Table 3). Artemisinin derivatives have been shown to induce reticulocytopaenia in preclinical studies, potentially by suppressing the erythroblasts [27]. Although the reticulocytes have not been reported in this study, the relatively moderate and transient drop of haemoglobin, noted mainly on day 7, and the increase of haemoglobin from day 14 to day 28 suggest that oral artemisinin derivatives therapy may not have late clinically relevant deleterious effect on haemoglobin.

Following the previous study reporting anaemia without haemoglobin evolution assessment [12], this study had an advantage of reporting haemoglobin evolution using a relatively large pooled malaria treatments database, including ACT, AS monotherapy and other non-artemisinin derivatives monotherapy, such as CQ, AQ and SP from various locations in Mali.

This study could not investigate the mechanisms involved in the haemoglobin evolutions observed. Indeed, the decrease on day 7 needs more study to improve the understanding of anaemia mechanisms, such as the reticulocytes evaluation. There is also a need for malaria treatment data from various countries, injectable AS treatment, vulnerable groups (pregnant women, HIV-infected subjects, patients with haemoglobinopathy, etc.).

## Conclusions

No association was found between ACT and severe delayed anaemia. Oral artemisinin derivative treatments for uncomplicated *P. falciparum* malaria are associated with a transient and clinically moderate haemoglobin decrease by day 7 but not associated with a delayed severe anaemia.

## Electronic supplementary material

Additional file 1: **Study treatment doses.** (PDF 107 KB)
