# Supplementary material for: Delayed anemia assessment in patients treated with oral artemisinin derivatives for uncomplicated malaria: a pooled analysis of clinical trials data from Mali
Source: Malar J. 2014 Sep 12;13:358. doi: 10.1186/1475-2875-13-358 (PMC4177171; doi:10.1186/1475-2875-13-358)
Supplement: Supplementary file 1 — Additional file 1: Study treatment doses. (PDF 107 KB) [file 12936_2014_3399_MOESM1_ESM.pdf]

Study Reference	Treatment information and doses
14	A single dose of 4 mg/kg Artesunate (Asunate Denk, Denk Pharma, Munich, Germany) was directly administered on the first day and a single dose of 2 mg/kg/day on each day thereafter. Doses were rounded up to the nearest quarter tablet. A full dose was re-administered in the case of rejection or vomiting in the first 30 minutes.
15	The Artemether-lumefantrine (AL) was Coartem (Novartis Pharma AG, Basel, Switzerland), given twice daily for 3 days to the following scheme: for patients < 15 kg, 1 tablet; 15–24 kg, 2 tablets; 25–34 kg, 3 tablets; ≥ 35 kg, 4 tablets.
16	Study drugs were given orally for 3 days (days 0, 1 and 2), dosed according to bodyweight. All doses were directly observed. Pyronaridine-artesunate was given once daily: ≥5 to <9 kg, one sachet; 9 to <17 kg, two sachets; 17 to <25 kg, three sachets (dose range 6.7/2.2 to 13.3/4.4 mg/kg/dose). Artemether-lumefantrine was given twice daily: ≥5 to <15 kg, one tablet; 15 to <25 kg, two tablets (dose range 1.3/8.0 to 4.0/24.0 mg/kg/dose); the second day-0 dose was 8 h after the first dose, the first day-1 dose was 24 h after the first day-0 dose, with all subsequent doses 12 h apart. Artemether-lumefantrine was given with food or milk as per local guidelines. Vomiting within

	30 minutes following the first drug dose resulted in re-dosing. Vomiting after repeat dosing or any subsequent dose resulted in study withdrawal and treatment with rescue medication (as per local guidelines).
17	Patients received either pyronaridine-artesunate once a day or artemether-lumefantrine twice a day, orally for three days (days 0, 1, and 2), plus respective placebo. Both treatments were given according to bodyweight (per dose for pyronaridine-artesunate: 20 kg to ≤25 kg one tablet, 26 kg to <45 kg two tablets, ≥45 kg to <65 kg three tablets, ≥65 kg to 90 kg four tablets, target 7·2/2·4 mg/kg to 13·8/4·6 mg/kg; per dose for artemether-lumefantrine: ≥20 kg to <25 kg two tablets, ≥25 kg to <35 kg three tablets, ≥35 kg to 90 kg four tablets). Food was not required for artemether-lumefantrine dosing to retain blinding. All doses were directly observed. Patients who vomited within 30 min could be re-dosed. If a patient vomited within 30 min of repeat dosing, he or she was withdrawn from the trial and given rescue medication (as per local clinical practice).
18	The dosages of Artesunate/Amodiaquine (Coarsucam™) were adapted according to the patient's body weight:<br>• Body weight ≥10 and <18 kg: daily dosage of 50/135 mg.<br>• Body weight ≥18 and <36 kg: daily dosage of 100/270 mg.

- Body weight ≥36 kg: daily dosage of 200/540 mg.

An interval of at least 8 hours must be maintained between the morning and the evening dose.

Coarsucam™ is administered in 1 or 2 intakes per day, according to randomisation. The treatment duration is 3 days.

The tablets are administered orally with a small amount of still drinking water. For the younger children, the tablets may be crushed and administered with still drinking water.

Artemether+lumefantrine (Coartem®):

The artemether/lumefantrine fixed-dose combination tablets contain 20/120 mg.

The dosages of Coartem® were adapted according to the patient's body weight:

- Body weight ≥10 and <15 kg: daily dosage of 40/240mg in 2 intakes
- Body weight ≥15 and <25kg: daily dosage of 80/480mg in 2 intakes
- Body weight ≥25 and <35 kg: daily dosage of 120/720mg in 2 intakes
- Body weight ≥35 kg: daily dosage of 160/960mg in 2 intakes

	An interval of at least 8 hours must be maintained between the morning and the evening dose.<br>The treatment duration is 3 days.<br>The tablets are administered orally with still drinking water.
19	All study medicines were administrated orally by designated study personnel at the health center. The Artesunate+Amodiaquine (AS+AQ) was Arsucam® coblister (AS 50 mg/AQ 153 mg; Sanofi-Aventis, Paris, France), given once daily for 3 days to the following scheme: for patients < 10 kg, 1/2 tablet AS + 1/2 tablet AQ; 10–20 kg, 1 tablet AS+ 1 tablet AQ; 21–40 kg, 2 tablets AS+ 2 tablets AQ; > 40 kg, 4 tablets AS+ 4 tablets AQ.<br><br>The Artesunate+Sulfadoxine-pyrimethamine (AS+SP) was Arsumax (AS 50 mg; Sanofi-Aventis, Paris, France) + sulfadoxine–pyrimethamine (S _ 500 mg/P _ 25 mg, Fansidar Roche, Burlington, NC), given once daily for 3 days to the following scheme: for patients ≤ 10 kg, 1/2 tablet AS+ 1/2 tablet SP; 11–20 kg, 1 tablet AS+ 1 tablet SP; 21–40 kg, 2 tablets AS+ 2 tablets SP; > 40 kg, 4 tablets AS+ 3 tablets SP. The SP is given only the first day, whereas AS is given over 3 days.<br><br>The Artemether-lumefantrine (AL) was Coartem (Novartis Pharma AG, Basel, Switzerland), given twice daily for 3 days to the following

	scheme: for patients < 15 kg, 1 tablet; 15–24 kg, 2 tablets; 25–34 kg, 3 tablets; ≥ 35 kg, 4 tablets.
20	Treatment was administered according to body weight at the following doses: Amodiaquine: 10 mg/kg/day from day 0 to day 2; Artesunate: 4 mg/kg/day from day 0 to day 2; SP: 25 mg/kg of sulfadoxine and 1.25 mg/kg of pyrimethamine in single dose on day 0. All drugs were administered directly by the study team and the child was observed for 30 minutes. If vomiting occurred before 30 minutes, the dose was repeated; for vomiting after 30 minutes, a half-dose was administered. In the case of persistent vomiting, the child was referred to a health center for rescue treatment with intramuscular or intravenous quinine and withdrawn from the study.
21	Artemether-lumefantrine was packaged in fixed-dose combination tablets, each containing 20 mg of artemether and 120 mg of lumefantrine. They were administered according to body weight (5–14 kg, one tablet; 15–24 kg, two tablets; 25–34 kg, three tablets; ≥ 35 kg, four tablets) in six consecutive doses: one dose was administered at enrollment, one dose 8 hours later, and then two doses on the second day after initiation of treatment. The Artesunate+Mefloquine (AS + MEF) treatment was supplied as two separate tablets in a single blister pack: a tablet of mefloquine and a tablet of artesunate. One dose was

	administered daily for three days. Dosage was determined by weight, with different blister packs for different weight arms. For a weight ≥ 31 kg, each blister contained three 200-mg tablets of artesunate plus three 250-mg tablets of mefloquine. For a weight of 15–30 kg, each blister contained three 100-mg tablets of artesunate plus three 125-mg tablets of mefloquine. For a weight of 10–14 kg, artesunate has been given at a dose of 4 mg/kg and mefloquine at a dose of 5 mg/kg.<br><br>For young children in both treatment arms, tablets were crushed and mixed with water. All drug doses were administered in the health center by a physician. A full dose was re-administered if a participant vomited the study drugs within 30 minutes of initial administration.
22	Artemether–lumefantrine was packaged in fixed-dose combination tablets each containing 20 mg of artemether and 120 mg of lumefantrine. They were administered according to body weight (5–14 kg, one tablet; 15–24 kg, two tablets; 25–34 kg, three tablets; ≥ 35 kg, four tablets) in four consecutive doses: one dose administered at enrollment, one dose eight hours later, and one dose on the first day and one dose on the second day after initiation of treatment.<br>The Artesunate plus Sulfamethoxypyrazine-pyrimethamine treatment was supplied as two separate tablets in a single blister pack. The tablets were color-coded for ease of identification: pink for

	sulfamethoxypyrazine-pyrimethamine and white for artesunate. One dose was administered daily for three days. Dosage was determined by weight, with different blister packs for different weight arms. For a weight ≥ 40 kg, each blister contained a 200-mg tablet of artesunate plus a 500/25-mg tablet of sulfamethopyrazine-pyrimethamine. A tablet of each drug was administered each day. For a weight of 20–39 kg, each blister contained a 100-mg tablet of artesunate plus a 250/12.5-mg tablet of sulfametho-pyrazine-pyrimethamine. A tablet of each drug was administered each day. For weights of 13–19 kg, 8–12 kg, and 5–7 kg, each blister contained a 50-mg tablet of artesunate plus a 125/6.25-mg tablet of sulfamethopyrazine-pyrimethamine. One and a half tablets, one tablet, and half of a tablet, respectively, of each drug were administered daily. For young children in both treatment arms, tablets were crushed and mixed with water. All drug doses were administered in the health center by a physician. A full dose was re-administered if a participant vomited the study drugs within 30 minutes of initial administration.
23	Treatment#1: Atesunate/Amodiaquine (50 mg/153 mg; Arsucam® from Sanofi-Aventis, Paris, France), AS 4 mg/kg/day + AQ 10 mg/kg/day, 3 days of treatment.<br>Treatment#2: Artesunate (50 mg; Arsumax® from Sanofi-Aventis) + Sulfadoxine/pyrimethamine (tablet of 500 mg/ 25 mg; from Roche). Artesunate 4 mg/kg/day for 3 days + Sulfadoxine/pyrimethamine 1

	tablet/20 kg, single dose administered on Day 0.<br>Treatment#3: Artesunate (50 mg; Arsumax® from Sanofi-Aventis), monotherapy, 4 mg/kg on Day 0 followed with 2 mg/kg/day on Days 1–4.
24	Chloroquine was administered according to body weight at the following doses: 10 mg/kg/day on day 0 and day 1, 5 mg/kg/day on day 2.
25	Sulfadoxine-pyrimethamine was administered as a single dose of 25 mg/kg of sulfaadoxine and 1.25 mg/kg of pyrimethamine on day 0.
26	Chloroquine was administered at 25 mg/kg over three days (10 mg/kg on days 0 and 1, 5 mg/kg on day 2).<br>Amodiaquine was administered at 25 mg/kg over three days (10 mg/kg on days 0 and 1, 5 mg/kg on day 2).<br><br>Sulfadoxine-pyrimethamine was administered as a single dose of 25 mg/kg of sulfaadoxine and 1.25 mg/kg of pyrimethamine on day 0.<br>All subjects were observed for 60 minutes to monitor for adverse reactions and to make sure that the medicine was not vomited. If vomiting occurred within 30 mn, the full dose was re-administered. If vomiting occurred after 30 mn a half dose was re-administered.
